# Development of Gender Non-Contentedness During Adolescence and Early Adulthood

**DOI:** 10.1007/s10508-024-02817-5

**Published:** 2024-02-27

**Authors:** Pien Rawee, Judith G. M. Rosmalen, Luuk Kalverdijk, Sarah M. Burke

**Affiliations:** 1 Department of Internal Medicine, University Medical Center Groningen, University of Groningen, Groningen, The Netherlands; 2 Interdisciplinary Center for Psychopathology and Emotion Regulation, Department of Psychiatry, University Medical Center Groningen, University of Groningen, Hanzeplein 1, 9713 GZ Groningen, The Netherlands

**Keywords:** Gender non-contentedness, Gender dysphoria, Adolescence, Sexual orientation

## Abstract

Adolescence is an important period for the development of gender identity. We studied the development of gender non-contentedness, i.e., unhappiness with being the gender aligned with one’s sex, from early adolescence to young adulthood, and its association with self-concept, behavioral and emotional problems, and adult sexual orientation. Participants were 2772 adolescents (53% male) from the Tracking Adolescents’ Individual Lives Survey population and clinical cohort. Data from six waves were included (ages 11–26). Gender non-contentedness was assessed with the item “I wish to be of the opposite sex” from the Youth and Adult Self-Report at all six waves. Behavioral and emotional problems were measured by total scores of these scales at all six waves. Self-concept was assessed at age 11 using the Global Self-Worth and Physical Appearance subscales of the Self-Perception Profile for Children. Sexual orientation was assessed at age 22 by self-report. In early adolescence, 11% of participants reported gender non-contentedness. The prevalence decreased with age and was 4% at the last follow-up (around age 26). Three developmental trajectories of gender non-contentedness were identified: no gender non-contentedness (78%), decreasing gender non-contentedness (19%), and increasing gender non-contentedness (2%). Individuals with an increasing gender non-contentedness more often were female and both an increasing and decreasing trajectory were associated with a lower global self-worth, more behavioral and emotional problems, and a non-heterosexual sexual orientation. Gender non-contentedness, while being relatively common during early adolescence, in general decreases with age and appears to be associated with a poorer self-concept and mental health throughout development.

**Supplementary Information:**

The online version contains supplementary material available at 10.1007/s10508-024-02817-5.

## Introduction

Gender identity refers to a person’s internal sense of being, male, female, neither, or both. When an individual’s gender identity does not match their birth-assigned sex, this may lead to significant psychological distress or impairment. This feeling of unease and distress is referred to as gender dysphoria. Gender dysphoria can for example be characterized by a strong desire to be of the other gender (or some alternative gender different from one’s assigned gender) and a strong desire to be rid of one’s primary and/or secondary sex characteristics (American Psychiatric Association, 2013). A broader concept is gender non-contentedness, referring to unhappiness with being the gender aligned with the birth-assigned sex (Egan & Perry, 2001; Potter et al., 2021). To illustrate the relation between these concepts; a young adolescent girl who mostly likes things seen as typical for boys and who dislikes the changes she goes through during puberty, might (temporarily) experience gender non-contentedness, although she might not experience gender dysphoria or wish to transition from female to male. Additionally, an individual who was assigned the male sex at birth, but experiences gender dysphoria and wishes to transition to female (i.e., a trans woman) can also experience gender non-contentedness.

An individual’s gender identity starts to develop in early childhood and is further established during adolescence with the onset of puberty and accompanying bodily changes (Peper et al., 2020). In an interview study with transgender youth, it became apparent that bodily changes due to puberty, social changes, and sexual exploration during adolescence were important factors in their feelings of unhappiness with their birth-assigned sex (Steensma et al., 2011).

The prevalence of gender non-contentedness in the general population has not been extensively studied yet, but data from the Netherlands, Taiwan, and the USA are available. In a Dutch general population sample of 941 children between the ages 11–19 in the year 1987, the Youth Self-Report (YSR) item “I wish to be of the opposite sex” with answer options never, sometimes or often was endorsed (sometimes or often) by around 5% of the children in all age categories. No significant differences per age category and sex were found (Verhulst et al., 1989). A recent study in a population sample of US American 10- to 11-year-olds found that around 9% of the children reported some degree of gender non-contentedness, as measured by the question “How much have you had the wish to be a < girl/boy > ?,” with 5 answer options ranging from rarely to always (Potter et al., 2021). Furthermore, in a Taiwanese sample (*n* = 1806) where gender contentedness was assessed with the question “Are you satisfied with your own gender?,” rated on a 4-point Likert scale from 1 (very dissatisfied) to 4 (very satisfied)), 14% reported dissatisfaction (very dissatisfied and dissatisfied) with their gender at one point during development (at age 13, or age 22, or both) (Kuo et al., 2021).

Regarding the prevalence of gender dysphoria in a more specific clinical setting, there has been a large increase in referral rates to gender clinics in the last decade in western countries (de Graaf et al., 2018; Wiepjes et al., 2018; Zucker et al., 2016). The exact reasons for this increase are not known, but it has been suggested that societal changes with improved recognition of transgender identities and more public awareness about gender services may play a role (Pang et al., 2020).

General population studies on the developmental trajectories of gender non-contentedness throughout adolescence are scarce. Existing population-based studies on gender non-contentedness in adolescence have been cross-sectional (e.g., Potter et al., 2021) or only contained one follow-up (e.g., Kuo et al., 2021). In the Taiwanese sample, 87% never reported any dissatisfaction, 8% reported dissatisfaction at age 13 but not at age 22, 5% at age 22 but not at age 13 and 1% reported persistent dissatisfaction.

Furthermore, the few longitudinal studies that have been conducted in a clinical setting found low persistence rates of early childhood gender dysphoric feelings into adolescence and adulthood (Ristori & Steensma, 2016; Singh et al., 2021). It was found that children who socially transitioned in early childhood were more likely to have persisting feelings of gender dysphoria (Steensma et al., 2013a). Most of these studies took place 20–50 years ago at a time when other diagnostic criteria applied or there was no formal diagnosis of gender dysphoria yet. Thus, these samples are likely quite heterogenic regarding the intensity of gender dysphoria and not all children might have met the criteria of a DSM-5 diagnosis. Additionally, the studies reported marked differences in persistence rates (2–39%; Ristori & Steensma, 2016; Singh et al., 2021). In a recent non-clinical sample of 317 children (mean age 8 years) who identified as transgender but were not assessed according to DSM-5 criteria for gender dysphoria, 94% still had a binary transgender identity and 4% had a non-binary identity at a 5-year follow-up assessment, at 13 years of age (Olson et al., 2022). In this sample, it was also suggested that a gender transition before puberty is associated with a continuing transgender identity. However, with only one follow-up assessment until age 13, their further gender identity development into mid- and late adolescence remains unknown. Also, without a DSM-5 gender dysphoria diagnosis, it is difficult to compare these recent findings to older longitudinal studies in a clinical setting.

In clinical samples, it was found that gender dysphoria is associated with a homosexual orientation in adulthood (Ristori & Steensma, 2016). Also, in population-based samples associations between gender-typical behavior in childhood and sexual orientation are found. For example, in one population-based study, children with gender non-conformity regarding play behavior at ages 3–5, were more likely to report a bisexual or gay sexual orientation later in life (Li et al., 2017). The association between gender variance and homosexual orientation in adulthood was reported to be less strong in general population samples compared to groups of individuals clinically referred for gender dysphoria (Steensma et al., 2013b).

Furthermore, a study found that children and adolescents referred for gender dysphoric feelings had a more negative self-concept compared to the standardization sample of the questionnaire. More specifically, youths with gender dysphoria more often had negative feelings about their bodies and had a lower global self-worth (Alberse et al., 2019; Rijn et al., 2013). In addition, prior studies in clinical samples of individuals with gender dysphoria found elevated rates of mental health problems (Dhejne et al., 2016), behavioral and emotional problems (de Vries et al., 2016), and autism spectrum disorders (Kallitsounaki & Williams, 2023).

Also, in a non-clinical sample of girls with a mean age of 8 years, a relationship between externalizing problems and gender non-contentedness (measured by the parent-report Child Behavior Checklist item, “Wishes to be of the opposite sex”) was found (van der Miesen et al., 2018). In another sample of 106 children from a US school with a mean age of 11 years, gender non-contentedness (measured by the gender contentedness scale of Egan & Perry, 2001) was associated with a lower global self-worth 1 year later but was not significantly associated with internalizing and externalizing problems (Yunger et al., 2004).

Thus, most studies conducted have focused on clinical (convenience) samples of children and adolescents who sought treatment at specialized gender clinics. However, not every individual that experiences gender non-contentedness will want to or is able to seek treatment (Renner et al., 2021). Therefore, the prevalence of such feelings in the general population and associations with the factors sex, sexual orientation, self-concept, and mental health are largely unknown. Moreover, longitudinal population-based studies with multiple follow-ups throughout adolescence and adulthood are lacking. Therefore, this project aimed to study the development of gender non-contentedness in a large, combined clinical and population cohort and to study which factors are associated with that.

## Method

### Preregistration

The current project has been preregistered on Open Science Framework (OSF) prior to data analysis https://osf.io/7n6hd. After registration, a few methodological adaptations were made: sexual orientation at assessment Wave 5 (age 22) was used instead of sexual orientation at Wave 6 (age 25), because there were less missing values at Wave 5. Furthermore, instead of using multiple logistic regressions, one multinomial logistic regression was performed to study associations between our independent variables and gender non-contentedness trajectories. Additional analyses were performed to study the association between gender non-contentedness trajectories and behavioral and emotional problems. Lastly, analyses regarding the association of gender non-contentedness with puberty stage and functional somatic symptoms were not included as these were beyond the scope of this article.

### Participants

We used data from the Tracking Adolescent’s Individual Lives Survey (TRAILS). Detailed information about TRAILS sampling procedures is provided elsewhere (Oldehinkel et al., 2015). In this study, data from both the population cohort (TRAILS) and the clinical cohort TRAILS-CC were used (combined: *N* = 2772; 53% male at T1) to enrich the sample for common child psychiatric diagnoses (see Table 1 for the sample characteristics).

**Table 1:** Descriptive statistics of demographic and questionnaire variables by TRAILS cohort type

TRAILS (*n* = 2229; 80%^a^)						
Female sex (%)	1131 (51%^b^)					
	T1	T2	T3	T4	T5	T6
Mean age (in years) (SD)	11.1 (0.6)	13.6 (0.5)	16.3 (0.7)	19.1 (0.6)	22.3 (0.7)	25.7 (0.6)
Number of individuals in the TRAILS sample that answered to the statement: "I wish to be of the opposite sex"	2172	2081	1654	1692	1496	1315
"I wish to be of the opposite sex" (%)	Never: 1904 (88%)	Never: 1948 (93%)	Never: 1571 (95%)	Never: 1646 (97%)	Never: 1453 (97%)	Never: 1277 (97%)
	Sometimes: 211 (10%)	Sometimes: 122 (6%)	Sometimes: 78 (5%)	Sometimes: 44 (3%)	Sometimes: 37 (2%)	Sometimes: 30 (2%)
	Often: 57 (2%)	Often: 11 (1%)	Often: 5 (0.3%)	Often: 2 (0.1%)	Often: 6 (0.4%)	Often: 8 (1%)
Self-concept—Physical appearance at T1 (SD)	3.12 (0.65)					
Self-concept—Global self-worth at T1 (SD)	3.33 (0.54)					
Sexual orientatio*n* at T5 (%) (*n* = 1495)					Heterosexual: 1386 (93%) Bisexual: 31 (2%) Homosexual: 78 (5%)	

TRAILS-CC (*n* = 543; 20%^a^)						
Female sex (%)	185 (34%^c^)					
	T1	T2	T3	T4	T5	T6
Mean age (in years) (SD)	11.1 (0.5)	12.9 (0.6)	15.9 (0.7)	19.1 (0.7)	22.0 (0.7)	26.1 (0.8)
Number of individuals in the TRAILS-CC sample that answered to the statement: "I wish to be of the opposite sex"	536	434	416	357	307	303
I wish to be of the opposite sex (%)	Never: 488 (91%)	Never: 396 (91%)	Never: 382 (92%)	Never: 331 (92%)	Never: 288 (94%)	Never: 282 (93%)
	Sometimes: 39 (7%)	Sometimes: 33 (8%)	Sometimes: 32 (8%)	Sometimes: 24 (7%)	Sometimes: 17 (5%)	Sometimes: 17 (6%)
	Often: 9 (2%)	Often: 5 (1%)	Often: 2 (0.5%)	Often: 2 (1%)	Often: 2 (1%)	Often: 4 (1%)
Self-concept—Physical appearance at T1	3.15 (0.69)					
Self-concept—Global self-worth at T1	3.23 (0.63)					
Sexual orientation at T5 (%) (*n* = 303)					Heterosexual: 271 (89%) Bisexual: 11 (4%) Homosexual: 21 (7%)	

^a^Of the total sample. ^b^Of the TRAILS sample. ^c^ Of the TRAILS-CC sample. TRAILS = the population cohort. TRAILS-CC = the clinical cohort. SD = standard deviation. Due to rounding, percentages might not add up to 100%

TRAILS is an ongoing prospective general population cohort study that started in the year 2000 and follows 2229 adolescents from the North of the Netherlands. The first measurements took place from March 2001 to July 2002 and participants’ age at the first Wave (T1) ranged from 10 to 12 years. Every two to three years, another data collection wave was conducted. TRAILS-CC is a clinical cohort (*N* = 543 at T1) that runs in parallel with TRAILS, using the same data collection methods. Participants from TRAILS-CC were recruited from a large child psychiatric outpatient clinic in the northern Netherlands with the same target area as covered by the population cohort. Children between 10 and 12 years of age, who had been referred to this clinic at any point in their life and regarding any type of mental health problem (thus a general clinical cohort, not specific for gender dysphoria), were eligible for participation in TRAILS-CC.

For the current study, the first six assessment Waves were used, covering the developmental period between late childhood (T1: 10–12 years of age) and early adulthood (T6: 24–26 years of age).

### Measures

#### Gender Non-Contentedness

Gender non-contentedness was assessed with the item "I wish to be of the opposite sex" of the Youth Self-Report (YSR; Achenbach & Rescorla, 2001) at T1 through T3 and with the same item of the Adult Self-Report (ASR; Achenbach & Rescorla, 2003) at T4 through T6. Participants indicated to which extent the statement applied to them during the past six months, by rating each item on a three-point Likert scale: 0-Not True, 1-Somewhat or Sometimes True, and 2-Very True or Often True.

#### Sexual Orientation

We used the question at T5 (mean age 22 years): “What do you think you are?,” with answer options heterosexual, homosexual, and bisexual as a measure of sexual orientation.

#### Self-Concept

Self-concept was measured by the Self-Perception Profile for Children (SPPC; Muris et al., 2003) at T1 (baseline). The SPPC evaluates self-perception relative to peers in five domains. In the current study, we used the “physical appearance” (Cronbach’s *α* = 0.81) and “global-self-worth” (*α* = 0.77) domains, because these were found to be associated with gender dysphoria in previous research (Rijn et al., 2013). These subscales both contain six statements and adolescents indicated on a 4-point Likert scale how much the statement applied to them (subscale score range 1–4, with 4 referring to positive self-concept or high global self-worth). The SPPC has good test–retest stability and regarding its validity, it was found that the SPPC correlates with other personality and psychopathology reports of parents, teachers and children (Muris et al., 2003).

#### Youth Self-Report/Adult Self-Report Total Problem Score

The total problem score of the YSR (T1 and T2; Cronbach’s *α* = 0.94, T3; *α* = 0.93) and the ASR (T4, T5 and T6; *α* = 0.96) was used as a measure of behavioral and emotional problems. Test–retest correlation after 8 days was 0.87 for the YSR total problem score (Achenbach & Rescorla, 2001) and the score appears as a reliable measurement of general psychopathology (Petot et al., 2023). The total problem score normally includes the item "I wish to be of the opposite sex,” but we calculated the score for every data wave excluding this item about gender non-contentedness. The mean score of 104 and 109 items was used as the total problem score of the YSR and ASR, respectively.

### Statistical Analyses

The statistical software R (Rstudio version 2021.09.0) was used for all statistical analyses. Chi-square tests were used to test if a relationship exists between gender non-contentedness and sex or cohort type (using individuals with complete data regarding the gender non-contentedness item). Wilcoxon signed rank test with Bonferroni correction were used to compare YSR/ASR total problem scores between gender non-contentedness trajectory groups (using individuals with complete data regarding gender non-contentedness and the total problem scores).

#### Developmental Trajectories of Gender Non-Contentedness

To identify subgroups (latent classes) based on the development of the wish to be of the opposite sex (an ordinal variable with three answer options), Latent Class Growth Analysis (LCGA) was performed on the entire sample of individuals who answered the item about the wish to be of the opposite sex at least once (*n* = 2766, combining TRAILS and TRAILS-CC; Lee et al., 2018). Since we used data with an ordinal outcome, the model was estimated with a threshold link function using the R-package lcmm. No random intercepts and slopes were included in the model (thus estimating a latent class growth model). Age in years (continuous variable) was used as the time variable. First, it was estimated if a linear or quadratic shape is most fitting to the data using a one-class solution. Then, models with one to six classes (the maximum number of subgroups we believed to have a meaningful interpretation) were estimated. Models ran 100 times with different start values (based on the initial one class model) to avoid local maxima (following guidelines described in; van de Schoot et al., 2016). The code used for this analysis is available on OSF. Several fit indices were used to select the best fitting trajectory shape and number of classes: Bayesian Information Criterion (BIC), Akaike Information Criterion (AIC), and a Lo-Mendell-Rubin Likelihood Ratio Test. A model with n classes was compared to a model with *n* + 1 classes based on these fit indices and the interpretability of the classes. Individuals that are not placed in a class were removed from further analysis. Individuals with a class probability (i.e., the chance of being placed in one class instead of another or none) below 0.75 were also removed (*n* = 419). This was done to exclude individuals with a gender non-contentedness trajectory that was largely deviating from the mean of the class.

#### Missing Data

After removing individuals with low class probability, multiple imputation was performed on the remaining sample, using the R-package mice (multivariate imputation by chained equations) to impute missing values. Data were imputed under the missing at random assumption. To assess the extent to which missing data affects the outcomes of the regression, a complete-case analysis was also performed regarding the factors associated with gender non-contentedness trajectories. Predictive mean matching was used for continuous variables, proportional odds model for ordinal variables, and polytomous logistic regression for the categorical variables, following guidelines described in (Buuren, 2018). See OSF for a further description of the multiple imputation process.

#### Factors Associated with Gender Non-Contentedness Trajectories

After multiple imputation, multinomial logistic regression was performed on the imputed datasets to test whether the variables sex, sexual orientation (dummy variables created for bisexual and homosexual), self-concept (subscales physical appearance and global self-worth) and cohort type (TRAILS or TRAILS-CC) were associated with gender non-contentedness. The gender non-contentedness classes defined by LCGA were compared to a class (also defined by LCGA) of individuals without gender non-contentedness. Results from the imputed datasets were pooled using the mice pooled function. These analyses were repeated on individuals with complete data.

As an additional post hoc analysis, we performed a Kruskal–Wallis test to analyze if individuals with gender non-contentedness at T1 differed in their scores on the physical appearance subscales at T1. Additionally, pairwise Wilcoxon rank tests were performed (with Benjamini–Hochberg as the *p*-value adjustment method) to see which gender non-contentedness trajectory groups differed from each other.

## Results

### Sample Characteristics

In Table 1, an overview of the sample characteristics can be found.

### Prevalence of Gender Non-Contentedness across Adolescence

**Fig. 1:**
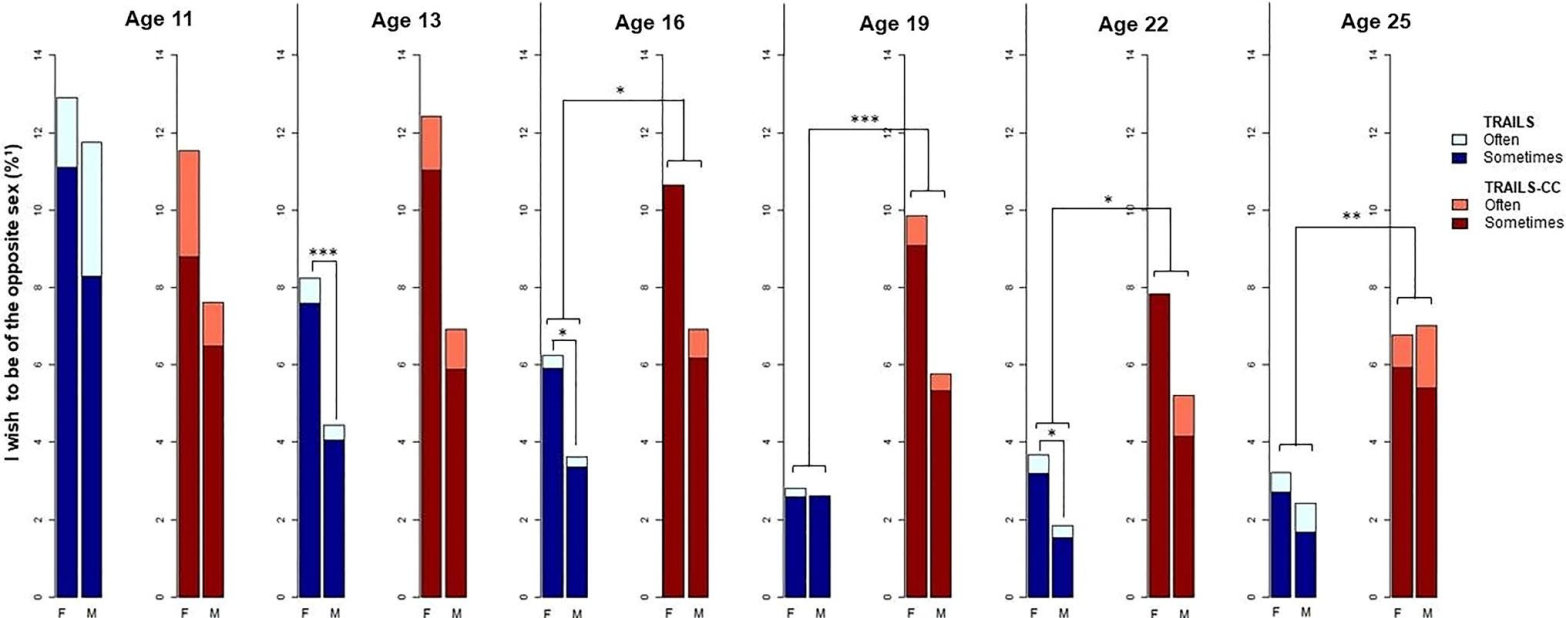
Prevalence of gender non-contentedness in both cohorts over time, distributed by sex assigned at birth. ^1^Percentages refer to the percentage of females or males reporting gender non-contentedness of the total female or male sample of TRAILS (*n* = 2229) or TRAILS-CC (*n* = 543). Age refers to the mean age of the assessment wave. F = Female sex. M = Male sex. *p < .05 of the chi-square testing if a relationship exists between sex (male or female) and gender non-contentedness (dichotomized for the chi-square test as No (never) or Yes (sometimes or often).*** *p* < .001 of the chi-square test of sex and gender non-contentedness

As can be seen in Fig. 1, gender non-contentedness was most prevalent at T1 (age 10–12 years), and the prevalence decreased with age. The prevalence was significantly higher in girls than boys at ages 13 (T2; *χ*^2^ = 13.91, *p* < 0.001) and 16 (T3; *χ*^2^ = 5.43, *p* < 0.05) in the TRAILS cohort. Also see Appendix A in Supplemantary Material for specific prevalence rates of gender non-contentedness per sex per timepoint. Gender non-contentedness prevalence was not significantly different in TRAILS and TRAILS-CC at T1 and T2, but it was significantly more prevalent in the clinical cohort at T3 (*χ*^2^ = 6.21, *p* < 0.05), T4 (*χ*^2^ = 18.68, *p* < 0.001), T5 (*χ*^2^ = 8.64, *p* < 0.05) and T6 (*χ*^2^ = 11.50, *p* < 0.01) (Table 1 and Fig. 1).

### Developmental Trajectories of Gender Non-Contentedness

**Fig. 2:**
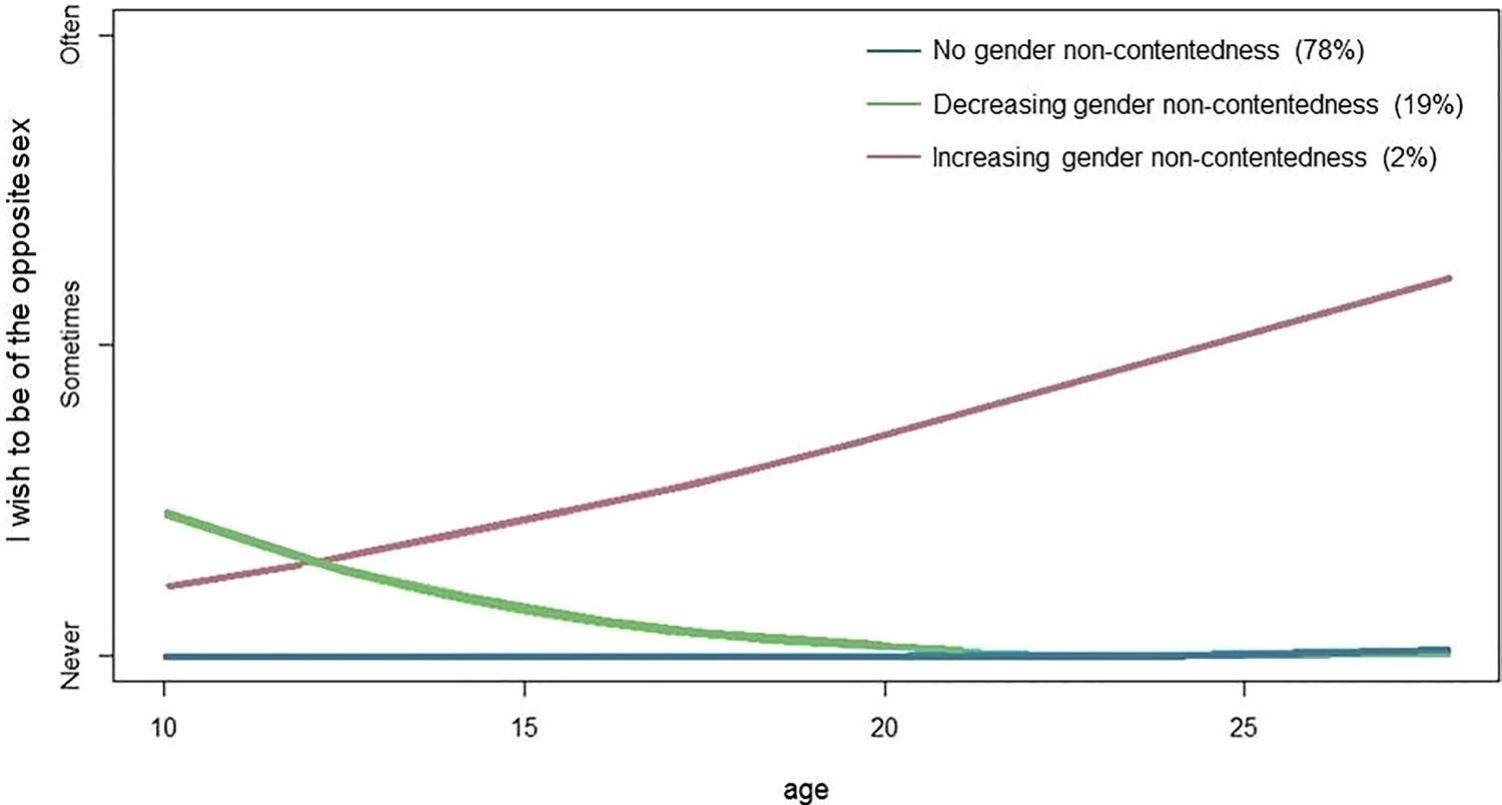
Trajectory groups of gender non-contentedness throughout adolescence and early adulthood, identified with latent class growth analysis. A mean trajectory line is plotted for every group. Percentages refer to the percentage of individuals in a trajectory group of the total sample after removal of individuals with a class probability < 0.75 and individuals not placed in a class. Due to rounding, percentages do not add up to 100%

LCGA was used to define gender non-contentedness trajectory types in the sample. For an overview of the model selection process and fit indices see Appendix B in Supplemantary Material. The three-class model was chosen because it had the lowest BIC value (5979.61) and did not contain classes with less than 1% of the sample (Fig. 2).

**Fig. 3:**
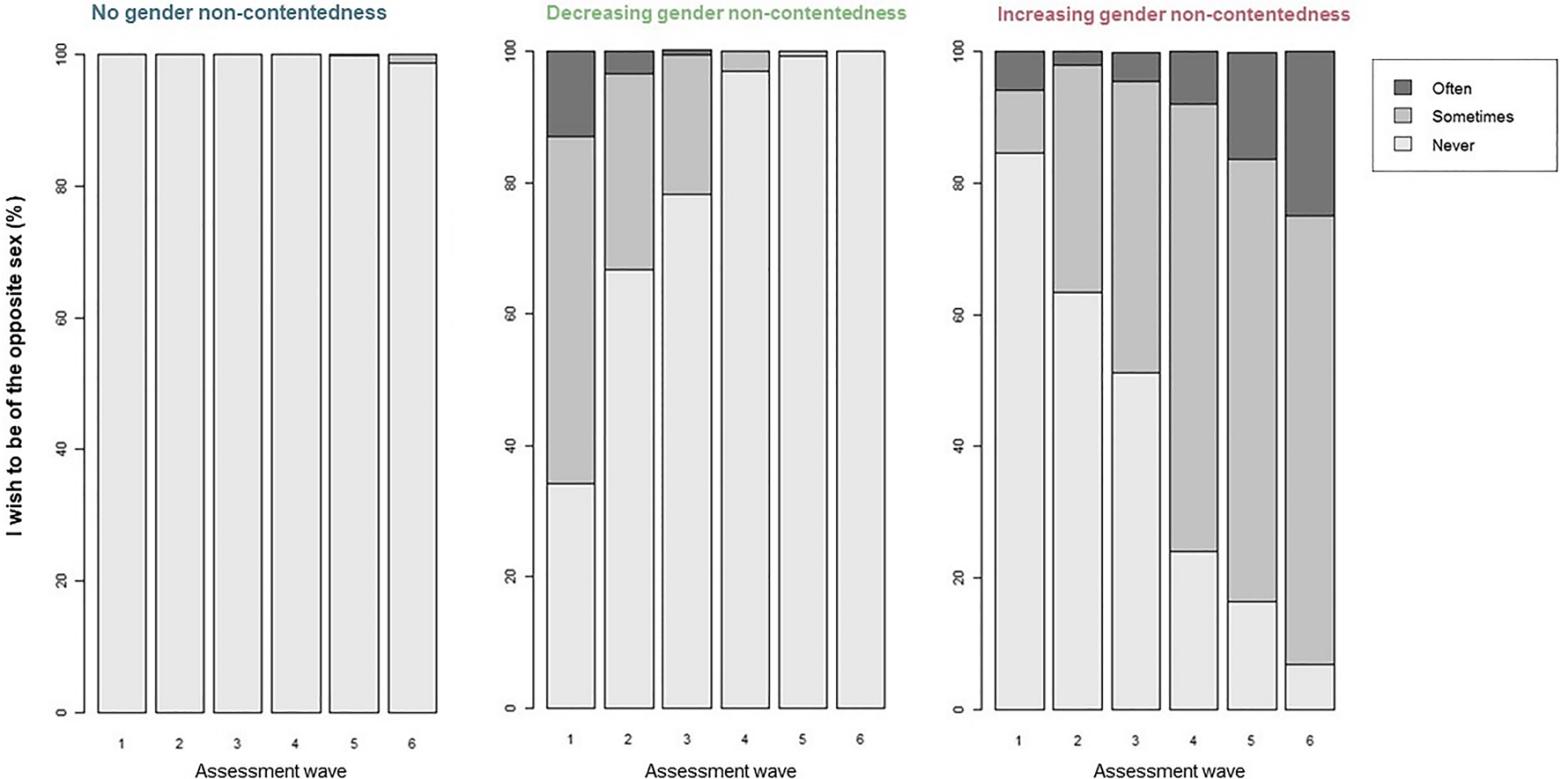
Distribution of gender non-contentedness within the trajectory groups. The percentage of individuals within each trajectory group that answered Often, Sometimes, or Never to the statement: “I wish to be of the opposite sex” is shown for the six different assessment waves

**Fig. 4:**
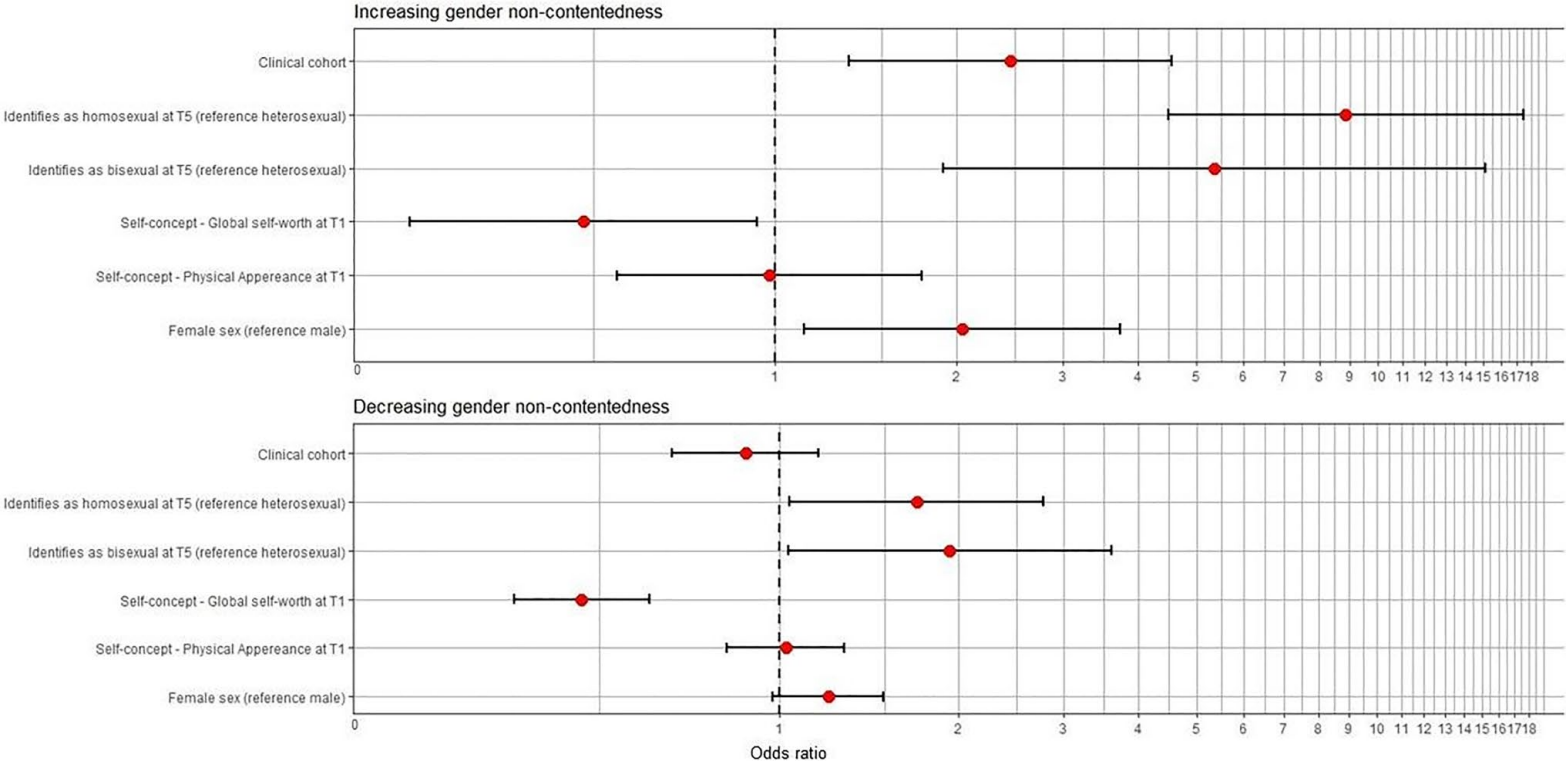
Odds ratios of having a decreasing or increasing gender non-contentedness trajectory instead of a trajectory without gender non-contentedness. The odds ratios can be interpreted as the odds of an individual of having an increasing or decreasing gender non-contentedness trajectory (instead of a trajectory without gender non-contentedness) depending on either their sex, self-concept or sexual orientation (while keeping the other predictor variables constant). Sex = female (reference male). Self-concept global self-worth and physical appearance: score between 1 and 4, measured at T1 (mean age 11). Sexual orientation is measured at T5 (mean age 22) with options: homosexual, bisexual and heterosexual (reference)

In Fig. 3, the mean trajectory line of each of the three trajectory groups is plotted. Most individuals followed a stable trajectory without any gender non-contentedness during adolescence and early adulthood (78% of individuals). Figure 4 shows the distribution of responses in the different trajectory groups to the statement: “I wish to be of the opposite sex” at the six assessment waves. As can be seen in Fig. 4, a few individuals who answered “sometimes” at the sixth assessment wave only, were also included in the group with a stable trajectory without gender non-contentedness.

The second largest group that was identified (19%) had a decreasing trajectory of gender non-contentedness (see Fig. 3). At the sixth assessment wave (around age 25), none of these individuals reported experiencing gender non-contentedness anymore (see Fig. 4).

A third group was found that had an increasing gender non-contentedness trajectory (2%). The majority of these individuals started reporting gender non-contentedness during adolescence with almost all of them reporting to sometimes or often experience gender non-contentedness around age 25 (see Fig. 4).

### Multiple Imputation

After trajectory class definition and removing individuals with a class probability < 0.75, multiple imputation was performed on the sample (*n* = 2347) to impute the remaining missing variables. A total of 70 multiple imputed datasets were created. For a further overview of the multiple imputation process, see OSF.

### Factors Associated with Gender Non-Contentedness

Using multinomial logistic regression, we analyzed how sex, self-concept (physical appearance and global self-worth subscales) at age 11, sexual orientation at age 22 and cohort type were related to having a gender non-contentedness trajectory (decreasing or increasing, compared to a trajectory without gender non-contentedness, see Fig. 3 for trajectory types). The odds ratios can be found in Fig. 4 and the exact results in Appendix C in Supplemantary Material. The odds ratios show the relative odds of an individual of having an increasing or decreasing gender non-contentedness trajectory (compared to having a trajectory without gender non-contentedness) depending on their characteristics (e.g., having the female sex).

An increasing gender non-contentedness trajectory was significantly associated with the clinical cohort, a homosexual or bisexual orientation, the self-concept subscale “Global Self-Worth,” and female sex, but not with the self-concept subscale “Physical Appearance.” A decreasing gender non-contentedness trajectory was also significantly associated with a homosexual or bisexual identification and global self-worth, but not with cohort type, sex, and the self-concept physical appearance subscale.

It was found that the odds of individuals to have an increasing or decreasing trajectory did not differ depending on an individual’s score on the physical appearance subscale (when also considering the other predictor variables). An additional post hoc Kruskal–Wallis test showed that at T1, the physical appearance self-concept subscale (H(2) = 30.697, *p* < 0.001) was significantly different between the answer groups of the gender non-contentedness item. Pairwise Wilcoxon rank tests showed that individuals who answered “sometimes” or “often” at T1 had significantly lower scores on the self-concept physical appearance subscale (referring to a more negative self-concept) compared to individuals who never experienced gender non-contentedness at T1 (“sometimes” compared to “never,” *p* < 0.001 and “often” compared to “never,” *p* < 0.05).

We found similar results when missing data were not imputed and only individuals with complete data were included in the multinomial logistic regression. The direction of effects was the same for all variables, but there were some differences in significance for the variables sex and sexual orientation. See Appendix D in Supplemantary Material, for more information about the complete-case analysis.

**Fig. 5:**
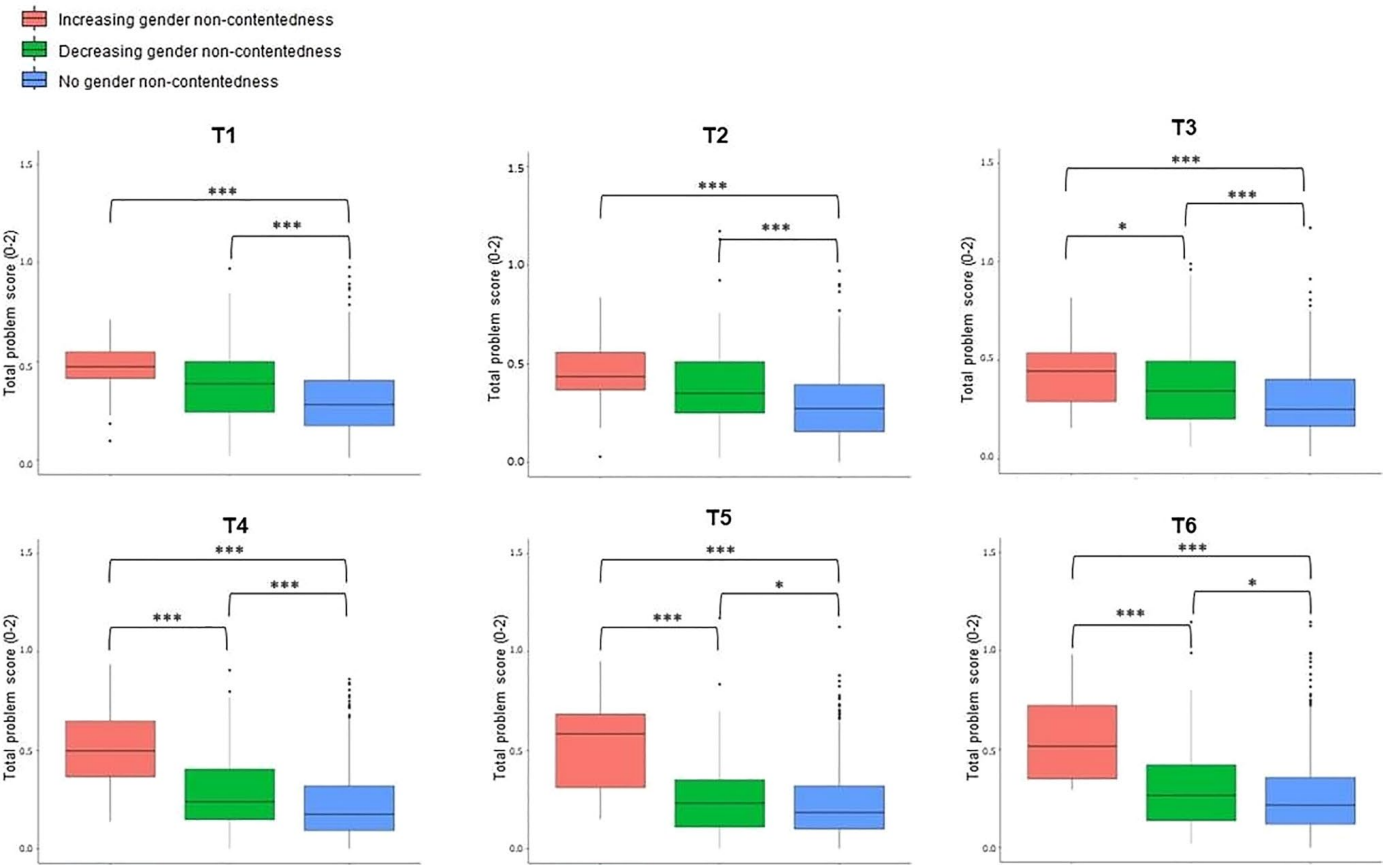
The total problem score of the YSR and ASR per gender non-contentedness trajectory group across the different timepoints. ***p < .001. *p < .05

In Fig. 5, the mean total problem score of the different trajectory groups across assessment waves can be found. Wilcoxon signed rank tests showed that individuals with an increasing and decreasing gender non-contentedness trajectory had a significantly higher total problem score than individuals without gender non-contentedness at any of the assessment waves (see Appendix C in Supplemantary Material, for the exact *p*-values). Furthermore, starting from T3 (i.e., mean age of 16 years), individuals with an increasing gender non-contentedness trajectory had a significantly higher total problem score than those with a decreasing gender non-contentedness trajectory.

## Discussion

We found that gender non-contentedness is most common around the age of 11 and that the prevalence decreases with age. Moreover, we identified three different developmental trajectory types of gender (non-) contentedness throughout adolescence and early adulthood: (1) the majority (78% of the sample) consistently indicated to never experience any gender non-contentedness, (2) a group reporting gender non-contentedness in early adolescence, but not any longer in adulthood (19% of the sample), and (3) a small group (2% of the sample) showing the opposite pattern of increasingly reporting gender non-contentedness with age. We found that female sex and participating in the clinical rather than population cohort was associated with increasing gender non-contentedness. In addition, individuals with increasing or decreasing gender non-contentedness trajectories had lower global self-worth, more behavioral and emotional problems, and more often had a homosexual or bisexual orientation compared to individuals without gender non-contentedness.

A major strength of our study is the 6-wave longitudinal design, which allowed us to model developmental trajectories of gender non-contentedness from late childhood through early adulthood (11–26 years). Furthermore, it was conducted in a combined general population and clinical sample, while most previous studies have reported on gender non-contentedness in samples of adolescents clinically referred for their gender identity problems, often including only a single follow-up assessment. Our study therefore provides more reliable epidemiological knowledge about the prevalence of gender non-contentedness among adolescents of the general Dutch population and provides new insights into the association with mental health problems.

A majority of adolescents (78%) indicated to never experience any gender non-contentedness. This is mostly in line with existing literature, which found that 87% of a sample of Taiwanese junior high school students were satisfied with their gender and that 90% of a sample of 9–10-year-olds from the Unites States never reported a wish to be of the other gender (Kuo et al., 2021; Potter et al., 2021).

In addition, in the current sample we found that a relatively large percentage (19%) of young adolescents reported gender non-contentedness in adolescence, but not in early adulthood. This is a larger percentage than found by Kuo et al.’s (2021) study, who reported a group (8% of 1806 children) who experienced gender dissatisfaction around age 13, but not anymore around age 22. The larger percentage of individuals with temporary, declining gender dissatisfaction in our sample might be explained by the wider age range (11–26 years) of the TRAILS sample. While we found a clear declining trend in gender non-contentedness with age in the TRAILS population cohort, such an age pattern was not observed in the cross-sectional data of an older (1987) Dutch sample of 941 children between ages 11 and 19, where no significant age differences were found (Verhulst et al., 1989).

Our study identified a group that started reporting gender non-contentedness in mid to late adolescence, showing an increasing trajectory into adulthood. In the literature about clinical gender dysphoria, a late-onset type is described, referring to the onset of gender dysphoric feelings after puberty (Lawrence, 2010). Furthermore, it has been noted that late-onset gender dysphoria may be preceded by or co-occur with various adjustment problems or psychopathology (Sevlever & Meyer-Bahlburg, 2019). Our findings confirm that gender non-contentedness also may start appearing after puberty.

Using LCGA, we did not identify a group with stable gender non-contentedness. A few individuals in the current sample quite consistently reported gender non-contentedness throughout adolescence and adulthood (*N* = 3, 0.1% of the total sample). However, this was not identified as a separate group given the small sample size.

Girls were more likely than boys to report gender non-contentedness at ages 13 and 16 (T2 and T3). Girls also had higher odds than boys to have an increasing trajectory of gender non-contentedness throughout adolescence (instead of a trajectory without gender non-contentedness). In an older (1987) Dutch general population sample, no significant sex differences in endorsement of the gender non-contentedness item was observed, although the prevalence was higher in adolescent girls at all ages (Verhulst et al., 1989).

Regarding clinical samples, it is known that referral rates to gender clinics are higher in adolescent girls than in boys (Aitken et al., 2015). A potential explanation offered in the literature for the current sex ratio in referral rates is that there might be less stigmatization of (birth-assigned) girls who behave masculine than boys who behave feminine, making it easier for girls to articulate their opposite sex wish (de Graaf et al., 2018; Perry et al., 2019). Additionally, a potential explanation for the higher prevalence of gender non-contentedness in girls than in boys in the current sample could be that girls more often believe that being a boy would come with certain advantages than the other way around (Perry et al., 2019).

Importantly, we found that both the increasing and decreasing gender non-contentedness trajectory groups had lower global self-worth at age 11 compared to adolescents without gender non-contentedness. Earlier studies also found that children referred to gender identity clinics have a more negative self-concept compared to a Dutch norm sample, specifically in the physical appearance and global self-worth domains (Alberse et al., 2019; Rijn et al., 2013). Low global self-worth was found to be associated with having an increasing or decreasing gender non-contentedness trajectory throughout adolescence. In contrast to our hypothesis, we did not find a significant association between the physical appearance scores and trajectories of gender non-contentedness in the multinomial logistic regression analysis. However, in post-hoc analyses, we found that those adolescents experiencing gender non-contentedness “sometimes” and “often” at T1 had a more negative body image than adolescents who never experienced gender non-contentedness at T1. This finding is in line with a recent study reporting on body dissatisfaction in adolescents with gender incongruence (when an individual’s gender identity does not match their birth-assigned sex), which was associated with worse psychological functioning (Verveen et al., 2023). Thus, the current study shows that a relationship between gender non-contentedness and a negative self-concept is also found in a combined general population and clinical sample and not only in children referred to gender identity clinics.

Self-worth and self-esteem are important factors in the well-being and mental health of adolescents. Next to a more negative self-concept, we found that gender non-contentedness was more prevalent in the clinical cohort of TRAILS than in the population cohort (at 4 assessment waves, not at T1 and T2). In addition, individuals with an increasing trajectory of gender non-contentedness had significantly higher YSR/ASR total problem scores at all timepoints. Gender non-contentedness has previously been associated with mental health problems (Potter et al., 2021) and clinical gender dysphoria has been reported to co-occur with diverse psychiatric problems, such as depression and anxiety disorders, eating disorders, and autism spectrum disorder (Bechard et al., 2017; Dhejne et al., 2016; Donaldson et al., 2018; Holt et al., 2016). This may potentially be due to minority stress, discrimination and/or bullying (Pellicane & Ciesla, 2022; Tankersley et al., 2021). Future research should use more extensive measures of gender non-contentedness and gender identity to further elucidate developmental patterns of these concepts in relation to adolescent well-being and mental health. Relevant to mention is that the data included in the current study were collected between 2001 (first timepoint population cohort) and 2020 (sixth timepoint clinical cohort). Societal awareness and acceptance of trans- and non-binary gender identities has improved in certain areas in the world. Therefore, future studies should examine whether more recently collected data on gender non-contentedness associate differently with measures of self-esteem and mental health.

The main limitation of the current study is that we could not use a very fine-grained proxy for gender non-contentedness. Although the YSR and ASR are widely used instruments, the item “I wish to be of the opposite sex” is worded in a binary manner, thereby excluding any responses reflecting a non-binary gender identity. In addition, this single item, with only three response options, may not fully capture the broader concept of gender non-contentedness. In a previous study in youth, gender non-contentedness was assessed in a similar way, but with five instead of three response options; “never,” “rarely,” “sometimes,” “often,” or “always.” In that study, 9% of individuals answered one of the latter four, with 6% of them answering “rarely” (Potter et al., 2021). It can be speculated that a large percentage of individuals in our sample who answered to “sometimes” wish to be of the opposite sex, might have answered “rarely” if this answer option was provided. Therefore, our numbers may overestimate the prevalence of gender non-contentedness. Furthermore, this item is multi-interpretable, as positive endorsement may reflect the participants’ wish to have the opposite sex’s identity, but just as well their gender role characteristics.

The gender non-contentedness prevalence was highest at the first assessment wave (mean age 11), but no data before this age were available. Thus, based on the current sample we cannot conclude that the prevalence of gender non-contentedness during development peaks around age 11. Also, in our study, no data on gender identity were available, but in the current, eighth assessment wave of TRAILS information on participants’ gender identity is being collected. Future studies may therefore investigate whether those participants who experienced gender non-contentedness in adolescence identified as transgender in adulthood. Sexual orientation was also assessed in a sub-optimal manner, by asking whether a person identified as homosexual, bisexual, or heterosexual. However, such response options are multi-interpretable. It is not known if individuals who experience gender non-contentedness (thus potentially including transgender boys and girls), used their sex assigned at birth or gender identity as a reference. In the current eighth TRAILS assessment wave sexual orientation is measured in a more detailed way, using both continuous response scales of andro- and gynephilia, and an item with categorical response options.

Another limitation is the combination of the general and clinical population cohort for the latent class growth analysis, as these are different sample types. The cohorts were combined to enlarge the sample sizes of the trajectory group, thereby increasing the statistical power of further analysis. The choice for a three-class instead of a two-class solution was made based on model fit criteria (AIC, BIC and a Lo-Mendell-Rubin likelihood ratio test, see Appendix B in Supplemantary Material). The choice of three instead of a four-class solution was made because the four-class solution contained a group with only 8 individuals. However, this can be seen as a limitation as some of the fit indices (AIC and Lo-Mendell-Rubin likelihood ratio test, but not BIC) indicated a better fit with the four-class solution. Furthermore, due to the longitudinal nature of the TRAILS cohort study, some participants dropped out and/or had missing data. If only the complete data would be used for the analysis, this could lead to bias. It was for example found that more males than females had missing data for the gender non-contentedness item. Therefore, multiple imputation was performed on the dataset prior to logistic regression analysis. For a further discussion on this method and the results, see the LCGA and MI document on OSF. Finally, in the logistic regression analysis, the association between 6 predictors and the longitudinal gender non-contentedness trajectory group was studied, but no adjustment for multiple testing was performed. Findings should therefore be interpreted with caution.

The results of the current study might help adolescents to realize that it is normal to have some doubts about one’s identity and one’s gender identity during this age period and that this is also relatively common. Furthermore, the insight that gender non-contentedness is relatively common during early adolescence in a general population and youth psychiatric care sample, might provide some perspective to clinicians primarily seeing individuals with intense gender dysphoric feelings and give them a more comprehensive view on the range of developmental patterns of gender identity in the general population and in children receiving youth psychiatric care.

In summary, having the wish to be of the opposite sex is relatively common in this combined general population and clinical sample. Our data indicate associations between experiencing gender non-contentedness and a poorer self-concept and mental health throughout adolescence.

### Supplementary Information

Below is the link to the electronic supplementary material.

Supplementary file1 (DOCX 18 kb)

Supplementary file2 (DOCX 151 kb)

Supplementary file3 (DOCX 9 kb)

Supplementary file4 (DOCX 17 kb)

## Data Availability

This study was preregistered at https://osf.io/7n6hd.

## Code Availability

Codes used for analyses can be found at https://osf.io/bcenj/?view_only=35226d2962f84d6f99cbea0af02b566b.

## References

[CR1] Achenbach, T. M., & Rescorla, L. A. (2001). *Manual for the ASEBA school-age forms & profiles.* ASEBA.

[CR2] Achenbach, T. M., & Rescorla, L. A. (2003). *Manual for the ASEBA adult forms & profiles*. ASEBA.

[CR3] Aitken M, Steensma TD, Blanchard R, Vanderlaan DP, Wood H, Fuentes A, Spegg C, Wasserman L, Ames M, Fitzsimmons CL, Leef JH, Lishak V, Reim E, Takagi A, Vinik J, Wreford J, Cohen-Kettenis PT, de Vries ALC, Kreukels BPC, Zucker KJ (2015). Evidence for an altered sex ratio in clinic-referred adolescents with gender dysphoria. Journal of Sexual Medicine.

[CR4] Alberse AE, de Vries ALC, Elzinga WS, Steensma TD (2019). Self-perception of transgender clinic referred gender diverse children and adolescents. Clinical Child Psychology and Psychiatry.

[CR5] American Psychiatric Association (2013). Diagnostic and statistical manual of mental disorders.

[CR6] Bechard M, VanderLaan DP, Wood H, Wasserman L, Zucker KJ (2017). Psychosocial and psychological vulnerability in adolescents with gender dysphoria: A “proof of principle” study. Journal of Sex & Marital Therapy.

[CR7] Buuren van, S. (2018). *Flexible imputation of missing data*. Taylor & Francis Ltd. 10.1201/9780429492259

[CR8] de Graaf NM, Carmichael P, Steensma TD, Zucker KJ (2018). Evidence for a change in the sex ratio of children referred for gender dysphoria: Data from the Gender Identity Development Service in London (2000–2017). Journal of Sexual Medicine.

[CR9] de Vries ALC, Steensma TD, Cohen-Kettenis PT, VanderLaan DP, Zucker KJ (2016). Poor peer relations predict parent- and self-reported behavioral and emotional problems of adolescents with gender dysphoria: A cross-national, cross-clinic comparative analysis. European Child & Adolescent Psychiatry.

[CR10] Dhejne C, Van Vlerken R, Heylens G, Arcelus J (2016). Mental health and gender dysphoria: A review of the literature. International Review of Psychiatry.

[CR11] Donaldson AA, Hall A, Neukirch J, Kasper V, Simones S, Gagnon S, Reich S, Forcier M (2018). Multidisciplinary care considerations for gender nonconforming adolescents with eating disorders: A case series. International Journal of Eating Disorders.

[CR12] Egan SK, Perry DG (2001). Gender identity: A multidimensional analysis with implications for psychosocial adjustment. Developmental Psychology.

[CR13] Holt V, Skagerberg E, Dunsford M (2016). Young people with features of gender dysphoria: Demographics and associated difficulties. Clinical Child Psychology and Psychiatry.

[CR14] Kallitsounaki A, Williams DM (2023). Autism spectrum disorder and gender dysphoria/incongruence: A systematic literature review and meta-analysis. Journal of Autism and Developmental Disorders.

[CR15] Kuo JH, Carrera RA, Mulyani LC, Strong C, Lin YC, Hsieh YP, Tsai MC, Lin CY (2021). Exploring the interaction effects of gender contentedness and pubertal timing on adolescent longitudinal psychological and behavioral health outcomes. Frontiers in Psychiatry.

[CR16] Lawrence AA (2010). Sexual orientation versus age of onset as bases for typologies (subtypes) for gender identity disorder in adolescents and adults. Archives of Sexual Behavior.

[CR17] Lee TK, Wickrama KAS, O’Neal CW (2018). Application of latent growth curve analysis with categorical responses in social behavioral research. Structural Equation Modeling.

[CR18] Li G, Kung KTF, Hines M (2017). Childhood gender-typed behavior and adolescent sexual orientation: A longitudinal population-based study. Developmental Psychology.

[CR19] Muris P, Meesters C, Fijen P (2003). The Self-Perception Profile for Children: Further evidence for its factor structure, reliability, and validity. Personality and Individual Differences.

[CR20] Oldehinkel AJ, Rosmalen JGM, Buitelaar JK, Hoek HW, Ormel J, Raven D, Reijneveld SA, Veenstra R, Verhulst FC, Vollebergh WAM, Hartman CA (2015). Cohort profile update: The TRacking Adolescents’ Individual Lives Survey (TRAILS). International Journal of Epidemiology.

[CR21] Olson, K. R., Durwood, L., Horton, R., Gallagher, N. M., & Devor, A. (2022). Gender identity 5 years after social transition. *Pediatrics*, *150*(2). 10.1542/PEDS.2021-056082 10.1542/peds.2021-056082 PMC9936352 35505568

[CR22] Pang, K. C., de Graaf, N. M., Chew, D., Hoq, M., Keith, D. R., Carmichael, P., & Steensma, T. D. (2020). Association of media coverage of transgender and gender diverse issues with rates of referral of transgender children and adolescents to specialist gender clinics in the UK and Australia. *JAMA Network Open*, *3*(3). 10.1001/JAMANETWORKOPEN.2020.11161 10.1001/jamanetworkopen.2020.11161 PMC7388018 32721030

[CR23] Pellicane MJ, Ciesla JA (2022). Associations between minority stress, depression, and suicidal ideation and attempts in transgender and gender diverse (TGD) individuals: Systematic review and meta-analysis. Clinical Psychology Review.

[CR24] Peper JS, Burke SM, Wierenga LM, Lanzenberger R, Kranz SG, Savic I (2020). Sex differences and brain development during puberty and adolescence. Handbook of clinical neurology.

[CR25] Perry DG, Pauletti RE, Cooper PJ (2019). Gender identity in childhood: A review of the literature. International Journal of Behavioral Development.

[CR26] Petot D, Petot JM, Chahed M (2023). Is the Youth Self-Report total score a reliable measure of both a general factor of psychopathology and Achenbach’s eight syndromes? A cross-cultural study. Journal of Psychopathology and Behavioral Assessment.

[CR27] Potter A, Dube S, Allgaier N, Loso H, Ivanova M, Barrios LC, Bookheimer S, Chaarani B, Dumas J, Feldstein-Ewing S, Freedman EG, Garavan H, Hoffman E, McGlade E, Robin L, Johns MM (2021). Early adolescent gender diversity and mental health in the Adolescent Brain Cognitive Development study. Journal of Child Psychology and Psychiatry.

[CR28] Renner J, Blaszcyk W, Täuber L, Dekker A, Briken P, Nieder TO (2021). Barriers to accessing health care in rural regions by transgender, non-binary, and gender fiverse people: A case-based scoping review. Frontiers in Endocrinology.

[CR30] Ristori J, Steensma TD (2016). Gender dysphoria in childhood. International Review of Psychiatry.

[CR31] Sevlever M, Meyer-Bahlburg HFL (2019). Late-onset transgender identity development of adolescents in psychotherapy for mood and anxiety problems: Approach to assessment and treatment. Archives of Sexual Behavior.

[CR32] Singh D, Bradley SJ, Zucker KJ (2021). A follow-up study of boys with gender identity disorder. Frontiers in Psychiatry.

[CR33] Steensma TD, Biemond R, De Boer F, Cohen-Kettenis PT (2011). Desisting and persisting gender dysphoria after childhood: A qualitative follow-up study. Clinical Child Psychology and Psychiatry.

[CR34] Steensma TD, McGuire JK, Kreukels BPC, Beekman AJ, Cohen-Kettenis PT (2013). Factors associated with desistence and persistence of childhood gender dysphoria: A quantitative follow-up study. Journal of the American Academy of Child and Adolescent Psychiatry.

[CR35] Steensma TD, van der Ende J, Verhulst FC, Cohen-Kettenis PT (2013). Gender variance in childhood and sexual orientation in adulthood: A prospective study. Journal of Sexual Medicine.

[CR36] Tankersley AP, Grafsky EL, Dike J, Jones RT (2021). Risk and resilience factors for mental health among transgender and gender nonconforming (TGNC) youth: A systematic review. Clinical Child and Family Psychology Review.

[CR37] van de Schoot R, Sijbrandij M, Winter SD, Depaoli S, Vermunt JK (2016). The GRoLTS-checklist: Guidelines for reporting on latent trajectory studies. Structural Equation Modeling: A Multidisciplinary Journal.

[CR38] van der Miesen AIR, Nabbijohn AN, Santarossa A, VanderLaan DP (2018). Behavioral and emotional problems in gender-nonconforming children: A Canadian community-based study. Journal of the American Academy of Child and Adolescent Psychiatry.

[CR29] Van Rijn AB, Steensma TD, Kreukels BPC, Cohen-Kettenis PT (2013). Self-perception in a clinical sample of gender variant children. Clinical Child Psychology and Psychiatry.

[CR39] Verhulst FC, Prince J, Vervuurt-Poot C, de Jong J (1989). Mental health in Dutch adolescents: Self-reported competencies and problems for ages 11–18. Acta Psychiatrica Scandinavica.

[CR40] Verveen A, van der Miesen AIR, de Graaf NM, Kreukels BPC, de Vries ALC, Steensma TD (2023). Body image in adolescents with gender incongruence and its association with psychological functioning. International Journal of Environmental Research and Public Health.

[CR41] Wiepjes CM, Nota NM, de Blok CJM, Klaver M, de Vries ALC, Wensing-Kruger SA, de Jongh RT, Bouman MB, Steensma TD, Cohen-Kettenis P, Gooren LJG, Kreukels BPC, den Heijer M (2018). The Amsterdam Cohort of Gender Dysphoria Study (1972–2015): Trends in prevalence, treatment, and regrets. Journal of Sexual Medicine.

[CR42] Yunger JL, Carver PR, Perry DG (2004). Does gender identity influence children’s psychological well-being?. Developmental Psychology.

[CR43] Zucker KJ, Lawrence AA, Kreukels BPC (2016). Gender dysphoria in adults. Annual Review of Clinical Psychology.

