# Supplementary material for: Development of Gender Non-Contentedness During Adolescence and Early Adulthood
Source: Arch Sex Behav. 2024 Feb 27;53(5):1813–25. doi: 10.1007/s10508-024-02817-5 (PMC11106144; doi:10.1007/s10508-024-02817-5)
Supplement: Supplementary file 1 — Supplementary file1 (DOCX 18 kb) [file 10508_2024_2817_MOESM1_ESM.docx]

# Appendix A: Sex-specific answers to the gender non-contentedness item of the Youth and Adult self-report

|  | | **T1** | **T2** | **T3** | **T4** | **T5** | **T6** |
| --- | --- | --- | --- | --- | --- | --- | --- |
| **Female sex (n=1316)** | | Total: 1291 | Total: 1213 | Total: 1022 | Total: 1057 | Total: 958 | Total: 895 |
|  | "I wish to be of the opposite sex" (%) | Never: 1127 (87%) | Never: 1107 (91%) | Never: 952 (93%) | Never: 1018 (96%) | Never: 918 (96%) | Never: 862 (96%) |
|  |  | Sometimes: 139 (11%) | Sometimes: 97 (8%) | Sometimes: 67 (7%) | Sometimes: 36 (3%) | Sometimes: 36 (4%) | Sometimes: 28 (3%) |
|  |  | Often: 25 (2%) | Often: 9 (1%) | Often: 3 (0.3%) | Often: 3 (0.3%) | Often: 4 (0.4%) | Often: 5 (1%) |
| **Male sex (n=1456)** | | Total: 1417 | Total: 1302 | Total: 1048 | Total: 992 | Total: 845 | Total: 723 |
|  | "I wish to be of the opposite sex" (%) | Never: 1265 (89%) | Never: 1237 (95%) | Never: 1001 (96%) | Never: 959 (97%) | Never: 823 (97%) | Never: 697 (96%) |
|  |  | Sometimes: 111 (8%) | Sometimes: 58 (4%) | Sometimes: 43 (4%) | Sometimes: 32 (3%) | Sometimes: 18 (2%) | Sometimes: 19 (3%) |
|  |  | Often: 41 (3%) | Often: 7 (1%) | Often: 4 (0.4%) | Often: 1 (0.1%) | Often: 4 (0.5%) | Often: 7 (1%) |

Table A1. Sex-specific answers on the statement: “I wish to be of the opposite sex”.
