# Supplementary material for: Development of Gender Non-Contentedness During Adolescence and Early Adulthood
Source: Arch Sex Behav. 2024 Feb 27;53(5):1813–25. doi: 10.1007/s10508-024-02817-5 (PMC11106144; doi:10.1007/s10508-024-02817-5)
Supplement: Supplementary file 2 — Supplementary file2 (DOCX 151 kb) [file 10508_2024_2817_MOESM2_ESM.docx]

**Appendix B : LCGA class selection**

Below, the process of selecting the best fitting model is described. It was found that a linear trajectory fitted the data better than a quadratic trajectory (BIC linear one class model: 6471.74 and BIC quadratic one class model: 6495.17). In Table B1, an overview of the fit indices for models with one to six classes can be found. The BIC value indicated that the three class model was the best fitting model and the values of other fit indices indicated that the four or five class model was preferred. We selected the three class solution because the four and five class solutions resulted in a class with only 9 or 12 individuals.

Six individuals were not assigned to a class due to the absence of information about gender contentedness. Individuals with a class probability (i.e., the chance of being placed in one class instead of another) lower than 0.75 were removed before further analysis and figure plotting (419 individuals were removed, resulting in a remaining sample of 2347 individuals).

Also see Figure B1 for an overview of samples used for the different analyses.

| **No. Classes** | **BIC** | **AIC** | **Lo-Mendell-Rubin likelihood ratio test (n tested against n+1)** | **Entropy** | **Sample size class 1** | **Sample size class 2** | **Sample size class 3** | **Sample size class 4** | **Sample size class 5** | **Sample size class 6** |
| --- | --- | --- | --- | --- | --- | --- | --- | --- | --- | --- |
| 1 | 6471.73 | 6453.96 | - | 1,00 | 2766 (100%) | - | - | - | - | - |
| 2 | 6050.48 | 6014.94 | P < 0.001 | 0,84 | 2656 (96%) | 110 (4%) | - | - | - | - |
| 3 | 5979.61 | 5926.28 | P < 0.001 | 0,56 | 2218 (80%) | 60 (2%) | 488 (18%) | - | - | - |
| 4 | 5988.22 | 5917.11 | P = 0.002 | 0,64 | 485 (18%) | 56 (2%) | 8 (0.3%) | 2217 (80%) | - | - |
| 5 | 6012.07 | 5923.19 | P < 0.001 | 0,66 | 50 (2%) | 2207 (80%) | 57 (2%) | 440 (16%) | 12 (0.4%) | - |
| 6 | 6000.09 | 5893.43 | P = 0.998 | 0,48 | 2201 (80%) | 12 (0.4%) | 446 (16%) | 50 (1.8%) | 57 (2%) | 0 (0%) |

**Results Latent Class Growth Analysis**

*Table B1:* Results of the latent class growth analysis. Fit indices for models with one to six classes are given. No. Classes = number of Classes, indicating the estimated number of different trajectories of gender non-contentedness in the sample; BIC = Bayesian Information Criterion; AIC = Akaike Information Criterion; Entropy = the accuracy of classification of individuals into the different latent classes (0-1).

**Samples used for the analyses**

*
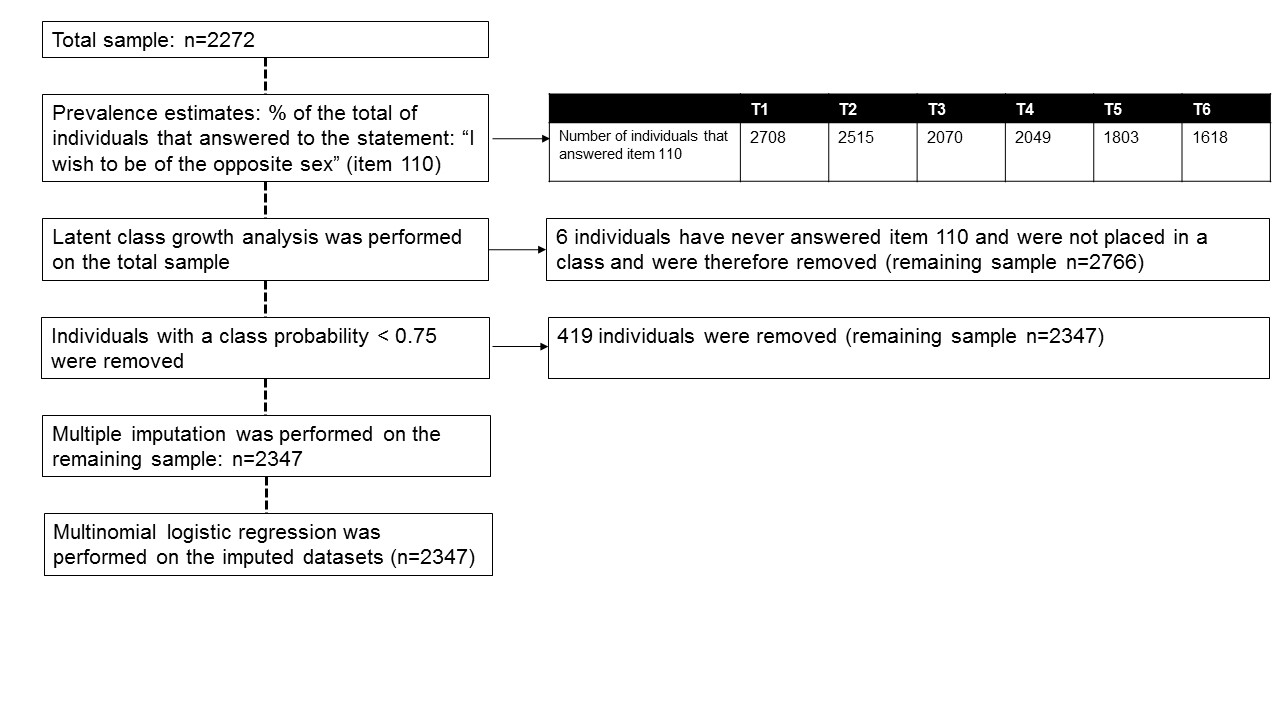
*

*Figure B1:* Overview of samples used for the different analyses
