# Supplementary material for: Development of Gender Non-Contentedness During Adolescence and Early Adulthood
Source: Arch Sex Behav. 2024 Feb 27;53(5):1813–25. doi: 10.1007/s10508-024-02817-5 (PMC11106144; doi:10.1007/s10508-024-02817-5)
Supplement: Supplementary file 3 — Supplementary file3 (DOCX 9 kb) [file 10508_2024_2817_MOESM3_ESM.docx]

# Appendix C: Exact results

In Table C1, the results of the multinomial logistic regression performed on the imputed datasets are shown. In Appendix D, the results of the complete case analysis can be found and the differences between these two analyses are colored red in Table C1. The directions of effect were the same in the analyses based on imputed data and the complete data.

| **Increasing gender non-contentedness** | | | **Decreasing gender non-contentedness** | | |
| --- | --- | --- | --- | --- | --- |
| **Variable** | **Odds ratio** | **p-value** | **Variable** | **Odds ratio** | **p-value** |
| Self-concept Appearance | 0.98 | 0.94 | Self-concept Appearance | 1.03 | 0.82 |
| Sex (female) | 2.04 | 0.020 | Sex (female) | 1.21 | 0.084 |
| Self-concept General | 0.48 | 0.031 | Self-concept General | 0.47 | < 0.001 |
| Bisexual orientation | 5.34 | 0.0015 | Bisexual orientation | 1.93 | 0.039 |
| Homosexual orientation | 8.86 | < 0.001 | Homosexual orientation | 1.70 | 0.035 |
| Cohort (clinical) | 2.46 | 0.0043 | Cohort (clinical) | 0.88 | 0.37 |

Table C1: Significant p-values are underlined. P-values that differed from the complete-case analysis regarding significance are colored red.

## Total problem score

Wilcoxon signed rank test with Bonferroni correction were used to compare YSR/ASR total problem scores between gender non-contentedness trajectory groups. In Table C2 the p-values of the pairwise comparisons per timepoint can be found.

| **T1** | | |
| --- | --- | --- |
|  | Increasing gender non-contentedness | Decreasing gender non-contentedness |
| Decreasing gender non-contentedness | 0.29 | - |
| No gender non-contentedness | < 0.001 | < 0.001 |
| **T2** | | |
|  | Increasing gender non-contentedness | Decreasing gender non-contentedness |
| Decreasing gender non-contentedness | 0.08 | - |
| No gender non-contentedness | < 0.001 | < 0.001 |
| **T3** | | |
|  | Increasing gender non-contentedness | Decreasing gender non-contentedness |
| Decreasing gender non-contentedness | 0.032 | - |
| No gender non-contentedness | < 0.001 | < 0.001 |
| **T4** | | |
|  | Increasing gender non-contentedness | Decreasing gender non-contentedness |
| Decreasing gender non-contentedness | < 0.001 | - |
| No gender non-contentedness | < 0.001 | 0.001 |
| **T5** | | |
|  | Increasing gender non-contentedness | Decreasing gender non-contentedness |
| Decreasing gender non-contentedness | < 0.001 | - |
| No gender non-contentedness | < 0.001 | 0.022 |
| **T6** | | |
|  | Increasing gender non-contentedness | Decreasing gender non-contentedness |
| Decreasing gender non-contentedness | < 0.001 | - |
| No gender non-contentedness | < 0.001 | 0.022 |

Table C2: P-values of Wilcoxon signed-rank tests of the difference in total problem scores between trajectory groups.
