# Supplementary material for: Development of Gender Non-Contentedness During Adolescence and Early Adulthood
Source: Arch Sex Behav. 2024 Feb 27;53(5):1813–25. doi: 10.1007/s10508-024-02817-5 (PMC11106144; doi:10.1007/s10508-024-02817-5)
Supplement: Supplementary file 4 — Supplementary file4 (DOCX 17 kb) [file 10508_2024_2817_MOESM4_ESM.docx]

# Appendix D: Complete-case analysis multinomial logistic regression

## Multinomial logistic regression

Complete-case analysis was used to see to what extent the missing data affect the outcomes of our multinomial regression analysis. In Table D1, an overview of odds ratios and p-values can be found. The directions of effects were the same as what was found with the imputed datasets for all variables. Regarding significance, a difference was found for the variables sex and sexual orientation. In the complete case analysis, sex is significantly associated with a decreasing gender non-contentedness trajectory and not with an increasing gender non-contentedness trajectory while it was the other way around in the analysis with the imputed datasets.

A bisexual orientation was significantly associated with a decreasing gender non-contentedness trajectory in the complete case analysis, but in the analysis with imputed datasets, a homosexual orientation was associated and not a bisexual orientation. The variables that differed regarding significance in the complete-case analysis had p-values slightly above or below 0.05 in the analysis with imputed datasets. Removal of individuals for the complete-case analysis apparently shifted this p-value slightly, causing different variables to be significant.

| **Increasing gender non-contentedness** | | | **Decreasing gender non-contentedness** | | |
| --- | --- | --- | --- | --- | --- |
| **Variable** | **Odds ratio** | **p-value** | **Variable** | **Odds ratio** | **p-value** |
| Self-concept Appearance | 0.91 | 0.77 | Self-concept Appearance | 0.98 | 0.88 |
| Sex (female) | 1.82 | 0.065 | Sex (female) | 1.42 | 0.0090 |
| Self-concept General | 0.49 | 0.047 | Self-concept General | 0.50 | < 0.001 |
| Bisexual orientation | 10.81 | < 0.001 | Bisexual orientation | 2.57 | 0.020 |
| Homosexual orientation | 10.38 | < 0.001 | Homosexual orientation | 1.64 | 0.058 |
| Cohort (clinical) | 2.57 | 0.005 | Cohort (clinical) | 1.02 | 0.90 |

Table D1. Results of the complete-case analysis. Significant p-values are underlined. P-values that differed from the analysis with imputed datasets regarding significance are colored red.
